# Analysis of factors influencing career preparation behaviors of Korean student athletes according to regional context

**DOI:** 10.3389/fpsyg.2025.1673966

**Published:** 2025-09-17

**Authors:** Seong Jun Ha

**Affiliations:** Department of Physical Education, Korea National University of Education, Cheongju, Republic of Korea

**Keywords:** student athletes, career preparation behavior, social support, school life adjustment, regional disparities, moderated mediating effect

## Abstract

This study analyzed the effects of social support and school adjustment on career preparation behaviors among Korean high school soccer players and empirically examined the moderating effect of region (metropolitan/non-metropolitan) on the relationship between these variables. To achieve this, a survey was conducted on 223 male high school football players from six high schools located in the Seoul metropolitan area and non-metropolitan areas. The relationships among school life adjustment, social support, and career preparation behaviors were analyzed using SPSS 27 and Process Macro 4.0 (Model 15). The results showed that both social support and school adjustment had a significant positive influence on career preparation behaviors, and the relationship between these variables was moderated by region. Specifically, social support directly influenced career preparation behaviors in the Seoul National Capital Area, but in the non-Seoul National Capital Area, it exerted an indirect influence through school adjustment. On the other hand, the influence of school life adjustment was relatively stronger in non-metropolitan areas. These results suggest that regional educational disparities structurally influence the career formation process of student athletes and emphasize the need for region-specific career support strategies.

## 1 Introduction

South Korean student-athletes tend to grow up with a strong sense of identity as “athletes” from an early age (Good et al., 1993), a tendency that is further reinforced by a centralized, performance-oriented athlete development system. As a result, student athletes are often placed in a structure where they have limited opportunities for academic participation or exploration of diverse career paths compared to general students. They also frequently face challenges in social adjustment or identity confusion due to unforeseen variables such as injuries, declines in performance, or early withdrawal from sports (Koukouris, 2001; Alfermann, 1995).

Especially during adolescence, there is a strong tendency to limit future aspirations to “athlete,” making it even more important to develop career planning skills that consider changeability and sustainability (Baillie and Danish, 1992). Career preparation is a developmental task closely related to long-term quality of life, and adolescence is a period when career exploration, planning, and decision-making abilities develop intensively (Savickas, 2005).

However, according to existing studies, athletes are more passive in career planning compared to general students and often fail to explore alternative careers after retirement or experience psychological maladjustment (Sowa and Gressard, 1983; Petitpas and Champagne, 1988). This is not simply due to a lack of opportunities but is closely related to the environmental and psychological constraints faced by student athletes.

In this context, social support is a key psychological and environmental resource for student athletes' career preparation. Emotional and informational support from parents, teachers, coaches, and peers promotes career exploration and provides stability and motivation (Lent et al., 2000). However, in Korea, there tends to be a quantitative and qualitative gap in social support due to low awareness and limited interest in activities outside of sports.

In addition, adjustment to school life serves as a foundation for student athletes to participate in academic activities and accumulate resources. Emotional belonging, academic achievement, and peer relationships are important factors in career choice, but in reality, student athletes find it difficult to enjoy the same school experience as other students due to competition schedules, training-centered timetables, and formal career education programs. As a result, their level of adjustment to school life declines, and their starting point for career preparation may be weakened.

The educational environment that influences career development shows structural disparities due to the uneven distribution of resources between regions. While the Seoul metropolitan area has abundant educational infrastructure, career information, college admission materials, and counseling resources, non-metropolitan areas are in poor conditions in terms of access to and quality of these resources (Kim et al., 2014; Park, 2018). In particular, sports infrastructure, teachers‘ career guidance capabilities, and parents' socioeconomic backgrounds vary across regions, and as a result, even if social support or school adjustment experiences are the same, their effects on career preparation behaviors may differ depending on the region.

Such regional disparities in career education go beyond a simple lack of resources and act as structural problems that hinder educational equity and equal opportunities. This study aims to analyze the effects of social support and school adjustment on career preparation behaviors among high school soccer players in Korea and empirically examine the moderating effects of regional context (metropolitan vs. non-metropolitan areas) on these relationships. The findings will provide practical implications for developing region-based, customized career support strategies to help student athletes balance athletic-focused careers with academic and life planning.

## 2 Theoretical background

### 2.1 Career preparation actions of student athletes

Career preparation is not merely a matter of choosing a job; it is a developmental task closely linked to the overall quality of life throughout one's lifetime (Savickas, 2005). Adolescence is a period of rapid development in career exploration, planning, decision-making abilities, and career preparation behaviors during this stage are considered important indicators of career maturity and adjustment levels in adulthood (Hartung et al., 2005). Therefore, career preparation behaviors during adolescence serve as a critical foundation for designing one's future life.

Athletes tend to be less active in career planning and educational activities compared to general students (Blann, 1985; Sowa and Gressard, 1983), which can lead to early career abandonment or psychological crises during career transition. In fact, former athletes often report difficulties in identity transition and maladjustment after retirement due to insufficient preparation for non-athletic situations (Kane, 1991; Chow, 2001).

Similar patterns are observed among student-athletes, particularly adolescent athletes who are prone to unexpected career transitions due to injuries or skill decline. Therefore, student athletes also require exploration and preparation for non-athletic career paths, which can be facilitated through systematic career education and social support. Guidance from key stakeholders such as teachers, coaches, and parents can play a crucial role in promoting career preparation behaviors among student athletes.

### 2.2 The importance of social support

Student-athletes are required to navigate the dual roles of academic learners and competitive athletes, balancing academic achievement with maintaining athletic performance. Academic underperformance, performance pressure, and career uncertainty that emerge from this duality can act as psychological stressors for student athletes, and these burdens can have a negative impact on their emotional stability and career planning. Social support is a key factor in mitigating such stress and enhancing student athletes‘ psychological resilience. Social support manifests in various forms, including emotional support, informational support, and instrumental support (Rees and Hardy, 2004), and the attention and support from key stakeholders such as parents, teachers, coaches, and peers have a substantial impact on enhancing student athletes' motivation for career exploration (Freeman and Rees, 2010).

When sufficient social support is provided, student-athletes can alleviate the burden of dual roles and establish a foundation for active participation in both academic and career activities. In particular, positive interactions with teammates and coaches can improve wellbeing levels and promote readiness for career transitions (Hagiwara et al., 2017). For these reasons, the establishment of a multidimensional social support system is essential to activate career preparation behaviors among student-athletes.

### 2.3 School life adjustment as career support

School life provides adolescents with a core environment for various developmental experiences, such as forming social relationships, achieving academic success, and developing self-identity. For student athletes, school is not just a place to attend classes, but also a place where they can access career information and experiment with their career possibilities through interaction with others. In this regard, adjustment to school life is an important variable that forms the basis for career behaviors.

However, in reality, student athletes have limited school experiences compared to general students due to factors such as competition schedules, training-centered timetables, and formal career education programs. This can lead to lower levels of school adjustment, which may weaken their identity as students and undermine the foundation for career preparation.

To address these issues, the concept of a “Dual Career Development Environment (DCDE),” which has been spreading recently in Europe, is gaining attention. This is a system that creates an environment where student athletes can pursue sports activities and academics simultaneously, supporting them in exploring various career possibilities. School adjustment functions as a central element in such a DCDE, providing the emotional and social foundation for career preparation behaviors.

### 2.4 Regional differences in career support

Theories explaining educational disparities have started with individual abilities and expanded their analytical framework to include cultural capital in the home, imbalances in educational resources, and differences in educational infrastructure between regions. In career education, region also acts as a key environmental factor, with significant differences in the quality and quantity of educational opportunities, access to career information, and social networks depending on the region (Amini and Nivorozhkin, 2015; Xiang and Stillwell, 2023).

In South Korea, metropolitan areas are concentrated with higher education institutions, career counseling agencies, and sports infrastructure, and teachers and parents tend to have higher career-related competencies. In contrast, non-metropolitan areas face structural issues such as an aging population, insufficient educational infrastructure, limited teacher resources, and poor access to career information (Kim et al., 2014; Lee et al., 2021).

Such regional disparities in career support limit the scope of career exploration that student athletes can experience and can lead to differing outcomes even when exposed to the same social support or school adjustment experiences. In other words, region can function as a key structural condition in the career formation process.

Therefore, analyzing how psychological and social factors influencing career preparation behaviors operate within regional contexts is crucial, as it can provide substantial evidence for establishing policy foundations to ensure educational equity and equal opportunities. This study aims to emphasize the necessity of region-specific career education strategies by empirically analyzing such regional moderation effects.

## 3 Research methods

### 3.1 Research subjects

South Korea's administrative divisions are divided into one special city, six metropolitan cities, one special self-governing city, eight provinces, and one special self-governing province. In South Korea, a special city is a city designated as the capital, and a metropolitan city is a city with a population of over 1 million. Metropolitan cities can be said to serve as economic and industrial centers. Provinces are regions composed of a mixture of cities (urban areas) and counties (rural areas). The Seoul National Capital Area refers to the capital city of Seoul and the surrounding areas of Incheon Metropolitan City and Gyeonggi Province. More than half of South Korea's total population is concentrated in this region, which is a hub of economic, cultural, and educational activities. This region constitutes the core of South Korea's educational and economic infrastructure, reflecting its position among the world's top 10 economies.

This study was conducted on male high school football players aged 16 to 18 in South Korea. In accordance with the purpose of this study, student athletes belonging to three teams in the Seoul National Capital Area and three teams in non-Seoul National Capital Area (rural regions) were selected as research subjects. A total of 115 student athletes (51.6%) from the Seoul National Capital Area and 108 student athletes (48.4%) from non-Seoul National Capital Area were selected as research subjects. The general characteristics of the study subjects are as follows: 95 (42.6%) were first-year students, 83 (37.2%) were second-year students, and 45 (20.2%) were third-year students. In terms of athletic experience, 86 (38.6%) had five years or less of experience, and 137 (61.4%) had six years or more of experience. Table 1 presents the distribution characteristics of the respondents.

**Table 1:** Characteristics of respondents.

	**Classification**	**Frequency (number)**	**Percentage (%)**
Area	Metropolitan area	115	51.6
	Non-capital region	108	48.4
Grade	First year	95	42.6
	Second year	83	37.2
	Third year	45	20.2
Sports career	5 years or less	86	38.6
	Over 6 years	137	61.4
Team achievements	Champion	92	41.3
	Runner-up	23	10.3
	Semifinals	32	14.3
	Quarterfinals and below	76	34.1

### 3.2 Scale

The questionnaire used in this study consisted of 70 items across four domains: background variables (region, grade, athletic experience, and team performance), school adjustment, social support, and career preparation behaviors. Background variables were measured using a nominal scale, while the remaining four domains were assessed using a 5-point Likert scale. The school adjustment scale and career preparation behaviors scale were developed to suit the educational characteristics and cultural background of Korean students.

The scale used to measure school adjustment was developed by Kim (2021) based on existing domestic and international scales related to school life, with modifications to suit the Korean context, to investigate the school adjustment of Korean students. This scale is divided into five sub-factors: ‘relationship with teachers,' ‘relationship with peers,' ‘understanding and participation in class,' ‘compliance with rules and activities,'and ‘online classes and communication,' and consists of a total of 50 items. In Kim (2021)'s study, the internal consistency of this scale ranged from 0.87 to 0.92. The reliability verification results ranged from 0.937 to 0.961.

To measure career preparation behaviors, we used the career preparation behaviors scale developed by Kim (1997) to suit Korean students. This scale is divided into three sub-factors: ‘information gathering behaviors,' ‘instrumental preparation behaviors,'and ‘practical effort,' and consists of a total of 18 items. In Kim (1997)'s study, the internal consistency of this scale was found to be 0.83 to 0.89. The reliability verification results were confirmed to be 0.859 to 0.918.

To measure social support, the Social Support Questionnaire for Transactions developed by Suurmeijer et al. (1995) was used. This scale is divided into five sub-factors: ‘socially related support,' ‘emotional support related to daily life,' ‘emotional support related to problems,' ‘instrumental support related to daily life,'and ‘instrumental support related to problems,' and consists of a total of 23 items. In the study by Suurmeijer et al. (1995), the internal consistency of this scale ranged from 0.38 to 0.76. In this study, after undergoing item analysis and review, it was determined that the ‘socially related support' factor alone sufficiently reflected the nature of social support, and thus it was analyzed as a single factor. The reliability verification resulted in a value of 0.92. Table 2 shows the reliability test results of the scales.

**Table 2:** Scale and reliability tests.

**Scale**		**Number of questions**	**Cronbach's α**
School Adjustment	Relationship with teachers	10	0.961
	Relationship with classmates	10	0.942
	Understanding and participating in class	10	0.937
	Compliance and Activities	10	0.948
	Online classes and communication	10	0.95
Social Support Questionnaire for Transactions (SSQT)	Items measuring Social Companionship (SC)	5	0.92
Career Preparation Behaviors	Information gathering behaviors	6	0.859
	Instrumental preparatory behaviors	5	0.883
	practical efforts	7	0.918
		66	

### 3.3 Data analysis

Research participants were informed of the purpose of the study, assured of anonymity and confidentiality, and obtained prior consent through their team leaders and parents. All research participants were provided with a detailed explanation of the study's purpose and methods, and they agreed to the anonymous processing of their data. It was explained that the participants' data would not be used for any purpose other than the research objectives. The survey was distributed from February 20 to April 20, 2024, and a total of 223 responses were collected.

The collected data were analyzed using SPSS 27 software. To verify the reliability of the data, internal consistency was estimated using Cronbach's α coefficient. Frequency analysis, descriptive statistics, and correlation analysis were conducted to identify the general characteristics of the research participants. Independent samples T-tests were conducted to verify the average differences between student athletes in different regions. To verify the moderated mediating effect, bootstrap analysis was conducted using the SPSS Process Macro 4.0 program developed by Hayes (2017). Bootstrap analysis was applied using model 15, with 5,000 samples extracted and a 95% confidence interval set. The significance of the mediating effect was determined to be significant if the 95% confidence interval did not include 0.

### 3.4 Research ethics

This study was conducted in accordance with the guidelines of the Helsinki Declaration. All research participants were provided with a detailed explanation of the purpose and methods of the study, and they consented to the processing of anonymized data. It was explained that participants' data would not be used for any purpose other than the research objectives. For student athletes under the age of 19, consent was obtained from their legal guardians. Personal information such as names, phone numbers, resident registration numbers, and addresses was not collected, so ethical approval was not required.

## 4 Research findings

### 4.1 Descriptive statistics and correlation coefficients

Table 3 shows the descriptive statistics and correlation analysis results for the main variables. School adjustment had a mean of 4.113, standard deviation of 0.6, and distribution of −0.146 to −0.886. Social support had a mean of 4.065, standard deviation of 0.733, and distribution of −0.218 to −1.11. Career preparation behaviors had a mean of 3.724, standard deviation of 0.785, and distribution of 0.067 to −0.645. The correlation coefficient between school life adjustment and social support was 0.634, between school life adjustment and career preparation behaviors was 0.606, and between social support and career preparation behaviors was 0.521.

**Table 3:** Descriptive Statistics and correlation coefficients.

**Analysis**		**School adjustment**	**Social support**	**Career preparation behaviors**
Correlation coefficient	School Adjustment	1		
	Social support	0.634^**^	1	
	Career preparation behaviors	0.606^**^	0.521^**^	1
Descriptive statistics	Average	4.113	4.065	3.724
	Standard deviation	0.6	0.733	0.785
	Skewness	−0.146	−0.218	0.067
	Kurtosis	−0.886	−1.11	−0.645

^**^ Correlation is significant at the 0.01 level (2-tailed).

### 4.2 Verification of average differences

Table 4 presents the results of a group comparison test conducted to understand the current status of high school student athletes in Korea and to identify the influence relationships and distributions among major control variables. In the analysis of group-level mean differences based on the activity regions of student athletes, significant differences were observed in school adjustment (*p* = 0.000), social support (*p* = 0.003), and career preparation behaviors (*p* = 0.003).

**Table 4:** Verification of average differences between groups.

**Variable**	**Group**	**School adjustment**		**Social support**		**Career preparation behaviors**	
		**Mean**	**SD**	**Mean**	**SD**	**Mean**	**SD**
Area	Metropolitan area	4.292	0.530	4.206	0.718	3.873	0.839
	Non-capital region	3.921	0.612	3.912	0.722	3.563	0.690
	*t*	4.846^***^		3.047^**^		2.996^**^	
Sports career	5 years or less	4.171	0.568	4.104	0.755	3.717	0.763
	Over 6 years	4.076	0.617	4.039	0.721	3.727	0.8
	*t*	1.149		0.645		−0.091	

^***^p < 0.001, ^**^p < 0.01, ^*^p < 0.05.

### 4.3 Verification of the modified moderated mediation model

To examine the moderating effect of region on the influence of social support and school adjustment on career preparation behaviors, we conducted an analysis following the procedure of Model 15 in the Process Macro for SPSS developed by Hayes (2017). The moderating effect was verified using bootstrapping, with a confidence interval of 95% and 5,000 samples. Social support had a significant positive effect on school adjustment (*B* = 0.62, *p* < 0.001).

The interaction term between social support and region had a significant effect on career preparation behaviors (*B* = −0.631, *p* < 0.001), and the increase in *R*^2^ due to the interaction term (Δ*R*^2^ = 0.035, *p* < 0.001) was also significant, indicating that region moderated the relationship between school adjustment and career preparation behaviors. The interaction term between school adjustment and region also had a significant effect on career preparation behaviors (*B* = 0.546, *p* < 0.01). The increase in *R*^2^ due to the interaction term (ΔR^2^ = 0.019, *p* < 0.01) was also significant, indicating that region moderated the relationship between social support and career preparation behaviors. Table 5 reports the results of the moderated mediation model. Table 6 displays the results of the direct and indirect effects.

**Table 5:** Results of the adjusted mediating effect model verification.

**Classification**		**Model 1 (DV: school adjustment)**			**Model 2 (DV: Career preparation behaviors)**		
		**Coefficient**	**SE**	***t***	**Coefficient**	**SE**	***t***
Constant		0.000	0.029	0.000	3.744	0.13	28.794^***^
IV	Social support	0.62	0.043	14.534^***^	1.205	0.282	4.266^***^
Mediator	School adjustment				−0.249	0.282	4.266
Moderator	Area				−0.011	0.083	−0.13
Interaction	social support × area				−0.631	0.168	−3.753^***^
	School adjustment × area				0.546	0.2	2.733^**^
Test of highest order interaction	ΔR^2^				0.035		
	*F*				14.087^***^		
	ΔR^2^				0.019		
	*F*				7.469^**^		
Model summary	R^2^	0.489			0.459		
	*F*	211.231^***^			36.763^***^		

^***^p < 0.001, ^**^p < 0.01, ^*^p < 0.05.

**Table 6:** Direct effects, indirect effects, and adjusted mediation indices.

**Direct effect (social support**→**school adjustment)**					
**Area**	**Effect**	**SE**	***t***	**LLCI**	**ULCI**
Metropolitan area	0.574	0.131	4.381^***^	0.316	0.832
Non-capital region	−0.057	0.105	−0.54	−0.264	0.151
**Indirect effect (social support**→**school adjustment**→**career preparation behaviors)**					
**Area**	**Effect**	**BootSE**	**BootLLCI**	**BootULCI**	
Metropolitan area	0.184	0.119	−0.029	0.434	
Non-capital region	0.522	0.059	0.392	0.625	
**Index of moderated mediation**					
**Area**	**Index**	**BootSE**	**BootLLCI**	**BootULCI**	
	0.338	0.127	0.062	0.564	

^***^p < 0.001, ^**^p < 0.01, ^*^p < 0.05.

95% Bootstrap LLCI.

95% Bootstrap ULCI.

**Figure 1:**
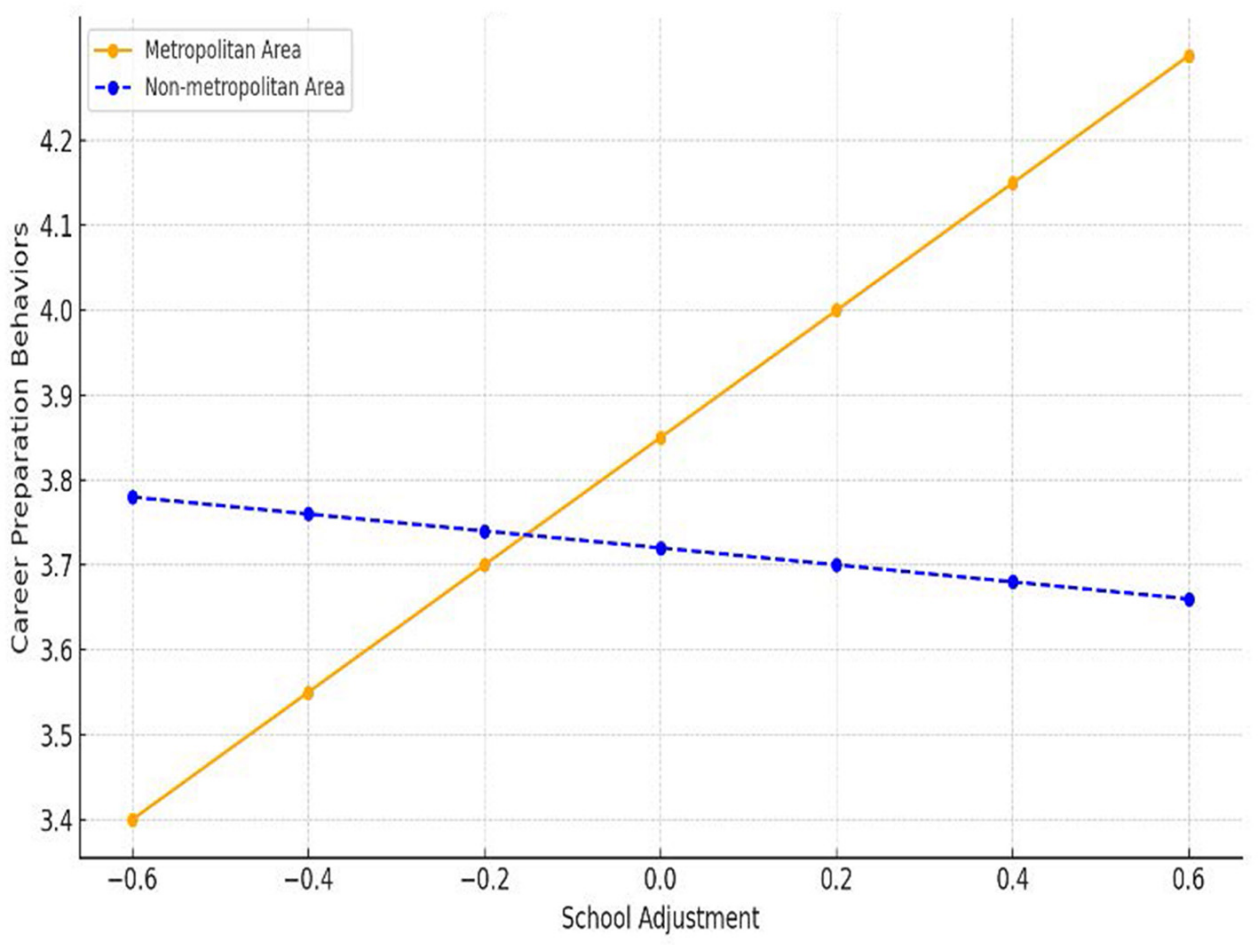
Moderating effect of region on the relationship between school adjustment and career preparation behaviors. In non-metropolitan areas, school adjustment has little or even negative influence on career preparation behaviors, whereas in metropolitan areas, the relationship is strongly positive.

**Figure 2:**
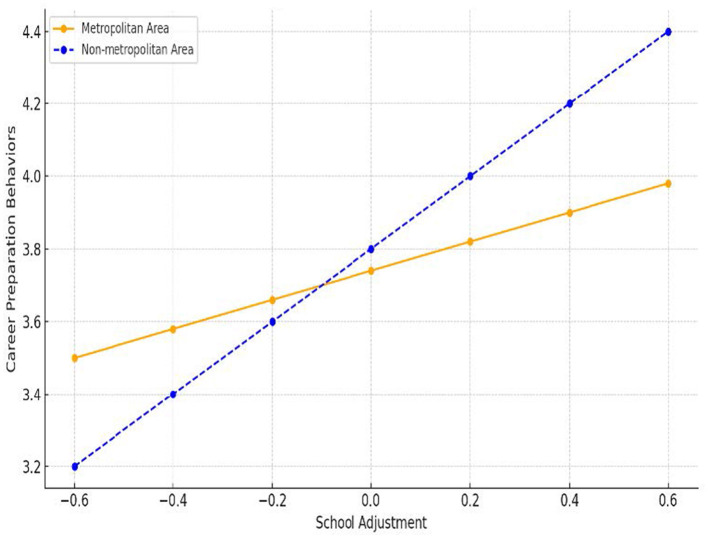
Moderating effect of region on the relationship between school adjustment and career preparation behaviors. In non-metropolitan areas, school adjustment has a stronger positive effect on career preparation behaviors compared to metropolitan areas.

The results of visualizing the moderating effects by dividing the regions into metropolitan and non-metropolitan areas are shown in Figures 1, 2. Figure 1 shows the moderating effects of region on the relationship between social support and career preparation behaviors. In the metropolitan area, career preparation behaviors increased as social support increased, but in the non-metropolitan area, the increase in career preparation behaviors was somewhat reduced when social support increased.

Figure 2 shows the moderating effects of region on the relationship between school adjustment and career preparation behaviors. In both the metropolitan and non-metropolitan regions, career preparation behaviors increased as school adjustment increased. However, the increase was relatively steeper in the non-metropolitan region than in the metropolitan region. These results show that the main factors influencing career preparation behaviors act differently depending on the regional and institutional context in Korea.

In particular, social support directly influenced career preparation behaviors in the Seoul National Capital Area, whereas its influence was limited in non-Seoul National Capital Areas. This is closely related to Korea's centralized education and career support structure. The Seoul National Capital Area offers relatively abundant access to diverse career information, programs, and alternative career exploration opportunities beyond sports, enabling student athletes to convert social support into more concrete career actions. On the other hand, non-metropolitan areas still have imbalances in career-related resources and opportunities, which limits social support to emotional support or prevents it from leading to career actions. This context suggests that future research should broaden its scope to include differences by gender and sport type, allowing for a more comprehensive exploration of how these factors influence career preparation behaviors.

Meanwhile, school adjustment had a stronger influence on student athletes in non-metropolitan areas. This suggests that Korean high school student athletes are generally structured to devote themselves to sports under an elite-centered training system, and that schools effectively function as the only career resource, especially in non-metropolitan areas where opportunities for career exploration and career education outside of sports are even more limited. Many high schools in Korea operate a special athletic talent program that limits participation in regular classes or provides only formal career education. In such an environment, students' emotional attachment to their schools, positive relationships with teachers and peers, and meaningful participation in classes become the starting point for career awareness and action. Considering the characteristics of Korean student athletes, who tend to make early career decisions and focus on a single goal (success as an athlete), adjustment to school life becomes a decisive factor in expanding the diversity and possibilities of their career paths.

**Figure 3:**
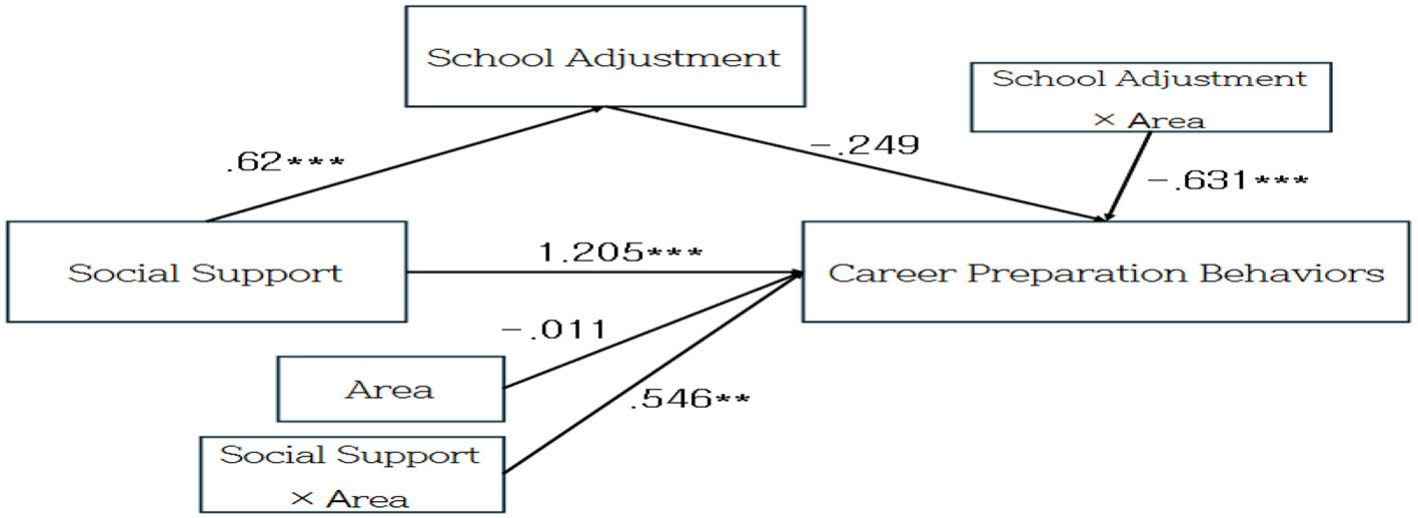
A moderated mediation model illustrating the effects of social support, school adjustment, and their interaction with region on career preparation behaviors. Social support indirectly influences career preparation behaviors via school adjustment, and both mediation and direct effects are moderated by regional context (metropolitan vs. non-metropolitan). Model 15 from PROCESS Macro was used. **p < 0.01, ***p < 0.001.

Furthermore, the patterns identified in this study were analyzed using a traditional measure of career preparation behaviors; therefore, different results may emerge if digital forms of social support (e.g., social networking services, online communities, and sports applications) or emerging career domains (e.g., sports media, esports, and sports rehabilitation) are taken into account. In particular, student-athletes in non-metropolitan areas may actively rely on digital-based support to compensate for insufficient offline resources, making this an important topic for future investigation. In addition, because this study employed a cross-sectional design, it was unable to capture temporal changes in these relationships; thus, longitudinal research is needed to examine how these dynamics evolve over time. Figure 3 presents the results of the moderated mediation model in a visualized form.

## 5 Conclusion

This study empirically examined the factors influencing career preparation behaviors among Korean high school soccer players, focusing on the roles of social support and school adjustment, and how their interaction differed by region. In particular, the study sought to clarify the structural relationships among psychological, social, and environmental factors surrounding student-athletes' career behaviors, with an emphasis on the contrasting contexts of metropolitan and non-metropolitan areas.

The findings revealed that both social support and school adjustment had significant positive effects on career preparation behaviors, but their influence varied by region. For student-athletes in metropolitan areas, social support exerted a direct influence on career preparation behaviors, whereas for those in non-metropolitan areas, social support tended to operate indirectly through the mediating role of school adjustment. Moreover, the effect of school adjustment was stronger in non-metropolitan areas, suggesting that schools function as a critical space for career preparation in regions where educational resources and career support systems are relatively limited.

These results highlight that the major factors shaping career preparation behaviors operate differently depending on the regional and institutional context of Korea. In metropolitan areas, abundant career resources and diverse opportunities provide a foundation for social support to be translated into concrete career actions. In contrast, in non-metropolitan areas, career-related resources and opportunities remain scarce, and social support often remains at the level of emotional encouragement rather than being transformed into actionable behaviors. To address this structural limitation, it is necessary to expand career information infrastructures tailored to non-metropolitan regions, strengthen school–community-linked career education programs, and provide practice-oriented career exploration activities such as mentoring and job shadowing.

This study is also significant in that it analyzed career preparation not only from the perspective of individual psychological traits but also from structural and environmental perspectives. Given that Korean student-athletes have long been constrained within a performance-oriented elite training system that has limited their opportunities for academic and career exploration, the present findings underscore the importance of considering social support and school adjustment together. By verifying the interactive and moderated mediating effects of these two factors, the study provides a more comprehensive explanation of the mechanisms underlying career preparation behaviors, with important implications for practice.

In sum, this study empirically confirmed the combined influence of social support and school adjustment in the career formation process of Korean high school student-athletes and identified regional moderation effects, thereby reinterpreting the direction of career support from a structural perspective. Furthermore, the findings strongly suggest the need for future research to expand this line of inquiry by examining differences across gender and sport types, incorporating digital forms of social support, adopting longitudinal designs to trace developmental changes, and including emerging career domains such as sports media, esports, and sports rehabilitation. Such expansions would provide a richer theoretical and practical foundation for career guidance systems that support student-athletes in designing sustainable and healthy lives beyond sports.

Nevertheless, this study has several limitations and directions for future research. First, the sample consisted only of male high school soccer players, which precluded an analysis of gender and sport-specific differences. As career preparation processes may vary according to gender identity and sport characteristics, future studies should include female, transgender, and athletes from diverse sports to enable more comprehensive comparisons across groups. Second, social support was measured only through traditional scales and did not capture digital forms of support, such as social networking services, online communities, or interactions through sports applications. Given the rapid changes in how adolescents acquire information and emotional resources, especially in non-metropolitan areas, future research should assess the frequency and effectiveness of digital social support and its role in regional disparities. Third, this study employed a cross-sectional design, which limited the ability to determine the causal order and temporal dynamics of the relationships among social support, school adjustment, and career preparation behaviors. As student-athletes' career behaviors may shift during transitional periods such as grade promotion or entry into higher education, future research should adopt longitudinal or cross-lagged designs to trace developmental changes more accurately. Fourth, the measure of career preparation behaviors used in this study focused mainly on traditional pathways (e.g., information seeking, preparatory actions) and did not adequately reflect emerging career options, such as sports media, esports, or sports rehabilitation. Future studies are therefore encouraged to develop and validate instruments that incorporate these emerging domains to better capture the evolving career landscape of student-athletes.

## Data Availability

The original contributions presented in the study are included in the article/supplementary material, further inquiries can be directed to the corresponding author/s.
